# Evolution of the Anther Gland in Early-Branching Papilionoids (ADA Clade, Papilionoideae, Leguminosae)

**DOI:** 10.3390/plants11070835

**Published:** 2022-03-22

**Authors:** Viviane Gonçalves Leite, Simone Pádua Teixeira, Ângela Lúcia Bagnatori Sartori, Vidal Freitas Mansano

**Affiliations:** 1 Departamento de Ciências Farmacêuticas, Faculdade de Ciências Farmacêuticas de Ribeirão Preto, Universidade de São Paulo (USP), Av. do Café, s/n., Ribeirao Preto 14040-903, Brazil; vianegleite@yahoo.com.br; 2 Instituto de Pesquisas Jardim Botânico do Rio de Janeiro, Diretoria de Pesquisa Científica, Rua Pacheco Leão, 915, Rio de Janeiro 22460-030, Brazil; vidal@jbrj.gov.br; 3 Centro de Ciências Biológicas e da Saúde, Laboratório de Botânica, Universidade Federal do Mato Grosso do Sul, Caixa Postal 549, Campo Grande 79070-900, Brazil; albsartori@gmail.com

**Keywords:** anther, anatomy, Angylocalyceae, Amburaneae, Dipterygeae, secretory structure

## Abstract

Papilionoideae is the most diverse subfamily of Leguminosae, especially in terms of floral morphology. The ADA clade shows some exciting floral features among papilionoids, such as anther glands. However, the evolution of the anther glands in such early-branching papilionoids remains unknown. Thus, we compared the occurrence, distribution, morphology, and evolutionary history of the anther glands in species of the ADA clade. Floral buds and/or flowers in 50 species were collected from herbarium specimens and investigated using scanning electron and light microscopy and reconstruction of ancestral character states. The anther apex has a secretory cavity, secretory duct, and phenolic idioblast. The lumen shape of the cavity and duct is closely related to the shape of the anther apex. The oval lumen is located between two thecae, the spherical lumen in the prominent anther apex and the elongated lumen in anthers with a long apex. The occurrence of cavities/ducts in the anther in only two phylogenetically closely related subclades is a unifying character -state. The floral architecture is not correlated with cavity/ducts in the anther but is possibly related to the type of pollinator. Future research needs to combine floral morphology and pollination systems to understand the evolution of floral designs and their diversification.

## 1. Introduction

Leguminosae is a species-rich family with more than 19,000 species distributed into about 765 genera [1]. The currently accepted classification of the family includes six subfamilies: Cercidoideae LPWG (12 genera/ca. 335 species), Detarioideae Burmeist., Handb. Naturgesch (84 genera/ca. 760 species), Dialioideae LPWG (17 genera/85 species), Duparquetioideae LPWG (1 genera/1 species), Caesalpinioideae DC (12 genera/ca. 335 species) and Papilionoideae DC (503 genera/ca. 14,000 species).

Among the Leguminosae subfamilies, Papilionoideae is considered the most diverse and ecologically successful [2], with a recent history of diversification during the Cenozoic [3,4]. Its diversity is expressed in floral morphology because the presence of papilionaceous flowers characterizes legumes, which also exhibit flowers with other architectural types see [5,6,7,8,9,10].

**Figure 1:**
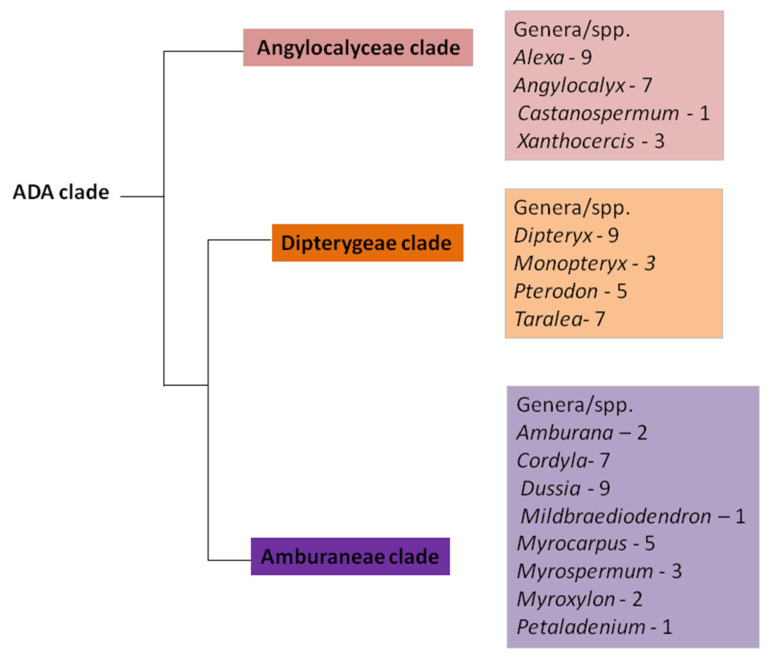
Phylogenetic relationships between the three subclades of the ADA clade modified from [1,10,12]. The Angylocalyceae subclade is a sister group to the Amburaneae and Dipterygeae subclades.

The early -branching papilionoids comprise plants with exciting flower morphology. They were included in the ADA clade and comprised about 74 species [11,12] (see Figure 1) distributed into three subclades: Amburaneae (eight genera), Angylocalyceae (four genera), and Dipterygeae (four genera) (Figure 1) [1,10,12]. Some members such as *Dipteryx alata* and *Pterodon pubescens* exhibit an unusual condition in the family, which is the presence of glandular appendages in the anther containing a secretory cavity. The secretory cavity consists of an isodiametric to elliptical lumen delimited by a uniseriate secretory epithelium and a parenchyma sheath [7,13]. It secretes sticky substances (oleoresins and polysaccharides) with a key role in plant reproduction, aggregating pollen grains and attaching them to the floral visitor’s body [13].

Among the groups within the ADA clade, the Dipterygeae subclade stands out by sharing the presence of secretory cavities and ducts on some parts of the plant body other than anthers, such as the bracteole, sepal, petal [7], pulvine, petiole, rachis [14], leaf [15,16], stem [17], fruit [18], and even early during plant development [19].

For groups closely related to Dipterygeae, there are no records of secretory ducts/cavities in the anther or other floral organs (see [5,8,9]). Interestingly secretory cavities have been found in the leaflets of *Cordyla*, *Myrocarpus*, *Myroxylon,* and *Myrospermum* [20,21], in genera of the Amburaneae clade, a sister group to the Dipterygeae subclade [1,10,12,22].

The absence of biological information for most species in the ADA clade is due to the sole use of surface analysis (scanning electron microscopy) in the study of flowers without including anatomical sections providing additional information concerning the internal anatomy and intra- and extracellular contents. 

In the current study, we present a detailed morphological and evolutionary investigation into the anther glandular appendages of the ADA clade species. We intended to (i) compare the occurrence, distribution and morphology, of the anther glandular appendages in the species of the ADA clade; (ii) to trace the evolutionary history of the secretory structures of the anther based on the recent phylogenetic hypothesis of the ADA clade (Figure 1) [1,10,12]; (iii) and to evaluate whether the presence of this condition is homologous in some groups.

The hypothesis we tested was that the presence of a secretory cavity/duct in the anther is widely distributed in the ADA clade, and thus, it was acquired by these taxa and can be considered as a synapomorphy for the group.

## 2. Results

Table 1 summarizes the results from our morphological analysis of the ADA clade species and other selected species. Additional information was obtained from the literature (Table 1). Because the presence of glands in the anther of this group is an uncommon condition, we analyzed and compared other characteristics related to the anther (for example, apex shape) and to other floral organs (for example, number per whorl and connation) in order to better understand their function in the flower and their evolutionary history.

### 2.1. Flower Morphology

**Figure 2:**
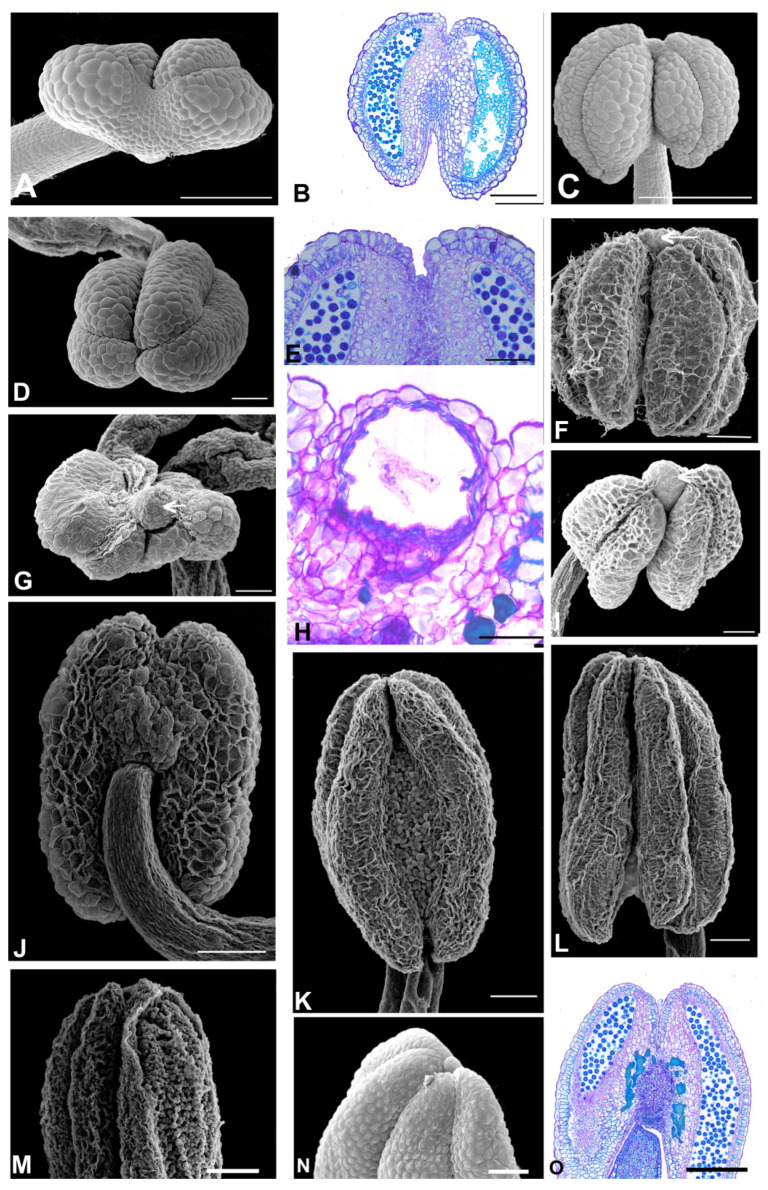
Aspects of the anther apex morphology in the species of the Amburaneae subclade. (**A**,**C**,**D**,**F**,**G**,**I**–**N**) = SEM, (**B**,**E**,**H**,**O**) = light microscopy (LM). (**A**,**B**) *Amburana acreana*. (**A**) Frontal view of the anther. Note that the anther apex is non-prominent. (**B**) Longitudinal section showing the anther. Note the absence of a secretory cavity between the two thecae. (**C**) *A. cearensis*. Adaxial view of the anther. Note that the anther apex is non-prominent. (**D**) A. erythrosperma. Frontal view of the anther. Note that the anther apex is non-prominent. (**E**) Longitudinal section showing the anther. Note the absence of a secretory cavity between the two thecae. (**F**) *Cordyla africana*. Adaxial view of the anther. Note that the anther apex is prominent (arrow). (**G**,**H**) *C. madasgascariensis*. (**G**) Frontal view of the anther. Note that the anther apex is prominent (arrow). (**H**) Longitudinal section showing the apex with a secretory cavity between the two thecae. (**I**) *C. haraka*. Frontal view of the anther. Note that the anther apex is prominent (arrow). (**J**) *Dussia discolor*. Abaxial view of the anther. Note that the anther apex is non-prominent. (**K**) *D. lehmanni*. Adaxial view of the anther. Note that the anther apex is non-prominent. (**L**) *D. macrophylla*. Adaxial view of the anther. Note that the anther apex is non-prominent. (**M**) *D. martinecensis*. Adaxial view of the anther. Note that the anther apex is non-prominent. (**N**,**O**) D. tesmanni. (**N**) Lateral view of the anther. (**O**) Longitudinal section showing the anther. Note the absence of a secretory cavity between the two thecae. Scale bars: (**A**,**B**,**N**), 200 μm; (**C**–**E**), 400 μm; (**F**–**O**), 100 μm; (**H**) 50 μm.

**Figure 3:**
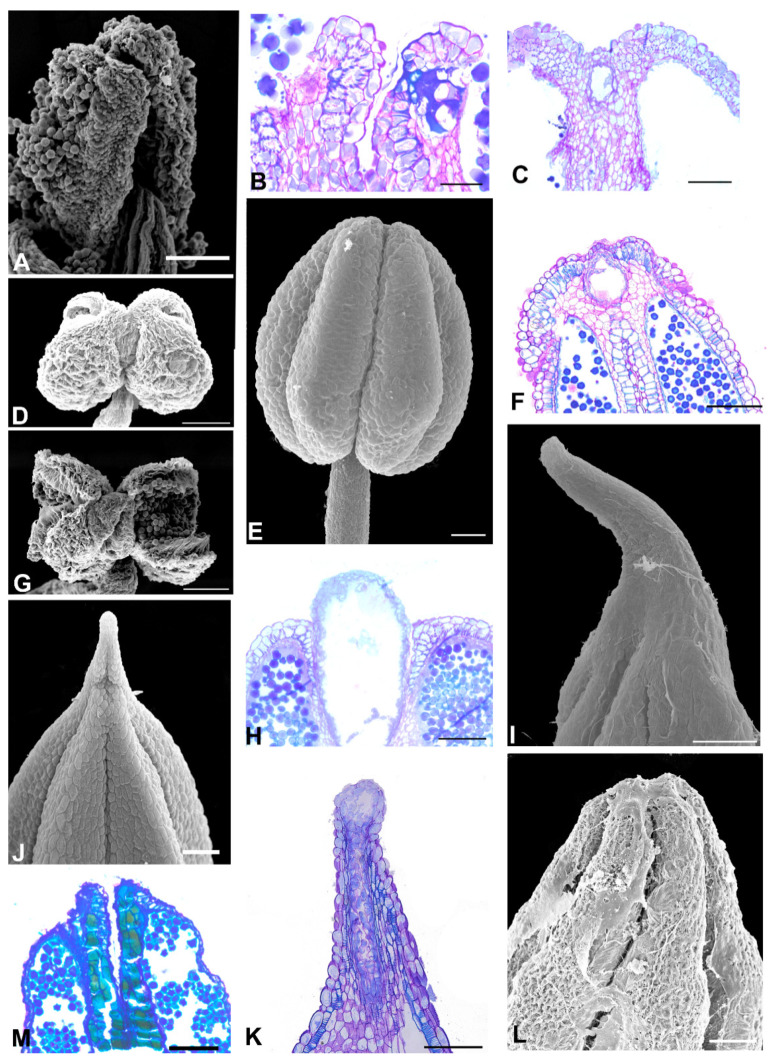
Aspects of the anther apex morphology in the species of the Amburaneae subclade. (**A**,**D**,**E**,**G**,**I**,**J**,**L**) = SEM, (**B**,**C**,**F**,**H**,**K**,**M**) = light microscopy, ML. (**A**,**B**) *Mildbraediodendron excelsum*. (**A**) Abaxial view of the anther. Note that the anther apex is non-prominent. (**B**) Longitudinal section showing the anther. Note the absence of a secretory cavity between the two thecae. (**C**) *Myrocarpus emarginatus.* Longitudinal section showing the anther apex with a secretory cavity. (**D**) *M. fastigiatus.* Frontal view of the anther. Note that the anther apex is non-prominent. (**E**,**F**) *M. frondosus.* (**E**) Adaxial view of anther. Note that the anther apex is non-prominent. (**F**) Longitudinal section showing the anther apex with a secretory cavity. (**G**,**H**) *Myrospermum frutescens*. (**G**) Frontal view of the anther. (**H**) Longitudinal section showing the anther apex with a secretory cavity. (**I**) *Myroxylon balsamum*. Lateral view of the anther. Note that the anther apex is prominent. (**J**,**K**) *M. peruiferum.* (**J**) Adaxial view of the anther. Note that the anther apex is prominent. (**K**) Longitudinal section showing the anther apex with a secretory duct. Note that the lumen shape of the secretory duct follows the shape of the anther apex, rounded in its apical portion (arrowhead) and elongated in the lower portion. (**L**,**M**) *Petaladenium urceoliferum*. (**L**) Adaxial view of the anther. Note that the anther apex is non-prominent. (**M**) Longitudinal section of the anther showing the phenolic cells. Scale bars: (**A**,**C**–**L**), 100 μm; (**B**), 50 μm.

The Amburaneae subclade species (Figure 2 and Figure 3) have flowers with a one-petalled corolla and free stamens (*Amburana* and *Mildbraediodendron*), no corolla and free stamens (*Cordyla*), a papilionaceous corolla and basally united stamens (*Dussia* and *Petaladenium*), a non-papilionaceous corolla (five equal petals) and basally united stamens (*Myrocarpus*), with a papilionaceous corolla and free stamens (*Myrospermum*), and a non-papilionaceous corolla (widely oval standard) and 10 free homogeneous stamens (*Myroxylon*). A prominent anther apex occurs in species of *Cordyla*, *Myrospermum*, *Myroxylon* (Figure 2F,G,I, and Figure 3G,I,J), and a non-prominent anther apex occurs in species of *Amburana*, *Dussia*, *Mildbraediodendron*, *Myrocarpus,* and *Petaladenium* (Figure 2A,C,D,J–N and Figure 3A,D,E,L).

**Figure 4:**
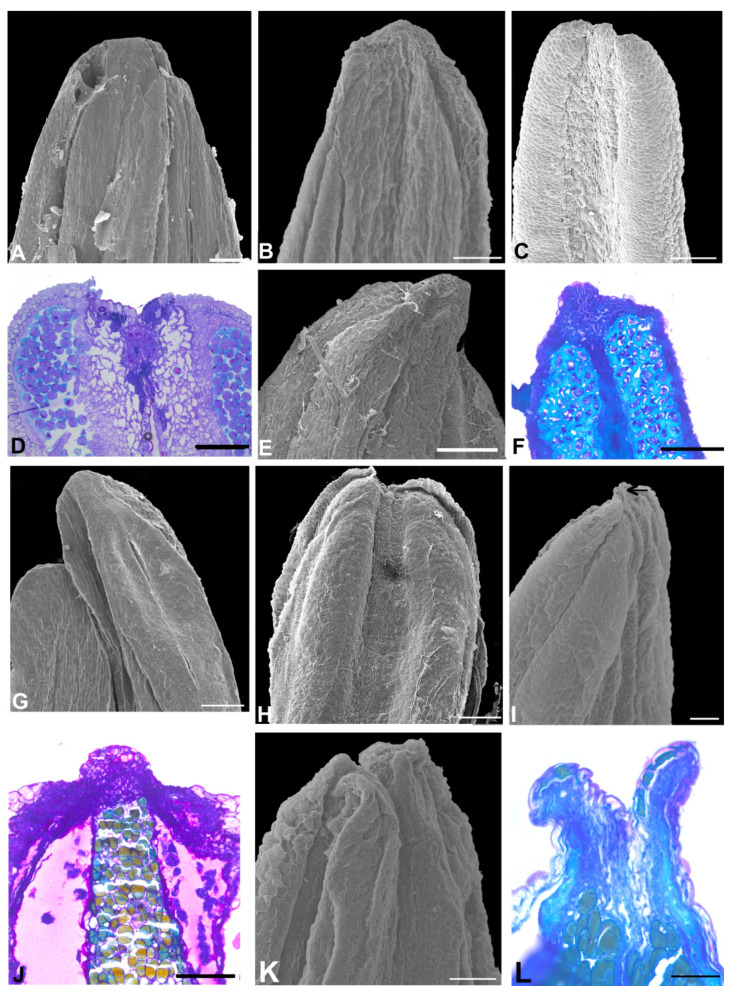
Aspects of the anther apex morphology in the species of the Angylocalyceae subclade. (**A**–**C**,**E**,**G**–**I**,**K**) = light microscopy, (**D**,**F**,**J**,**L**) = SEM. (**A**) *Alexa bauhiniflora*. Adaxial view of the anther. Note that the anther apex is non-prominent. (**B**) *A. canaracunensis*. Adaxial view of the anther. Note that the anther apex is non-prominent. (**C**,**D**) *A. cowanii*. (**C**) Lateral view of the anther. Note that the anther apex is non-prominent. (**D**) Longitudinal section of the anther showing the phenolic cells. (**E**,**F**) *A. grandiflora*. (**E**) Lateral view of the anther. Note that the anther apex is a small prominence. (**F**) Longitudinal section of the anther showing the phenolic cells. (**G**) *A. imperatrizes*. Adaxial view of the anther. Note that the anther apex is non-prominent. (**H**) *A. leiopetala*. Adaxial view of the anther. Note that the anther apex is non-prominent. (**I**,**J**) *A. superba*. (**I**) Lateral view of anther. Note that the anther apex is a small prominence. (**J**) Longitudinal section of the anther. Note the absence of a secretory cavity between the two thecae. (**K**,**L**) *A. wachenheimii*. (**K**) Lateral view of the anther. Note that the anther apex is non-prominent. (**L**) Longitudinal section of the anther showing the phenolic cells. Scale bars: (**A**,**C**,**D**), 200 μm; (**B**,**G**,**L**), 50 μm; (**E**,**H**), 200 μm; (**F**,**I**–**K**) 100 μm.

**Figure 5:**
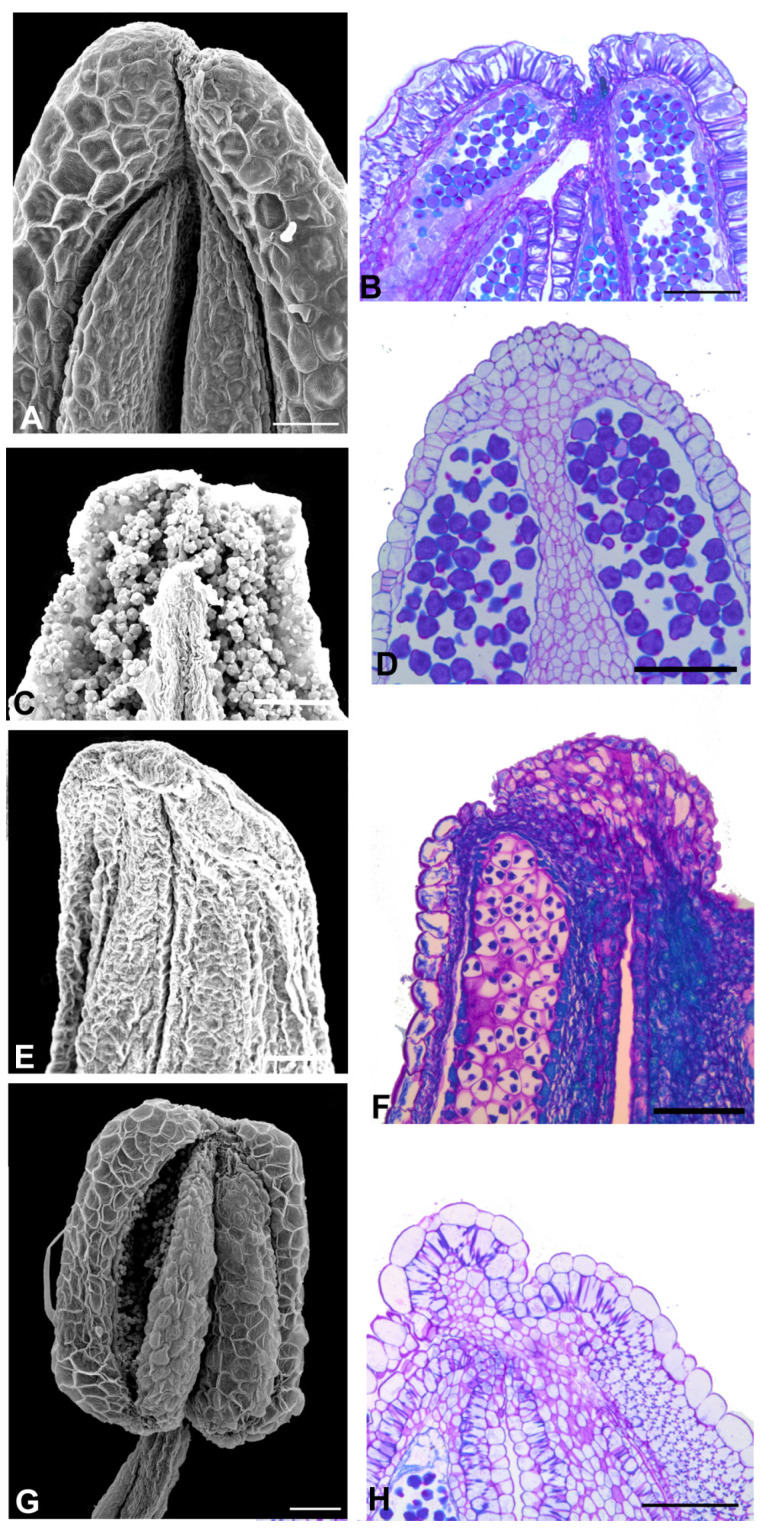
Aspects of the anther apex morphology in the species of the Angylocalyceae subclade. (**A**,**C**,**E**,**F**) = SEM, (**B**,**D**,**F**,**H**) = light microscopy, LM. (**A**,**B**) *Angylocalyx pynaertii*. (**A**) Adaxial view of the anther. Note that the anther apex is non-prominent. (**B**) Longitudinal section of the anther. Note the absence of a secretory cavity between the two thecae. (**C**,**D**) *A. talbotii*. (**C**) Lateral view of the anther. Note that the anther apex is non-prominent. (**D**) Longitudinal section of the anther. Note the absence of a secretory cavity between the two thecae. (**E**,**F**) *Castanospermum australe.* (**E**) Adaxial view of the anther. Note that the anther apex is a small prominence. (**F**) Longitudinal section of the anther. Note the absence of a secretory cavity between the two thecae. (**G**,**H**) *Xanthocercis zambesiaca*. (**G**) Adaxial view of the anther. Note that the anther apex is non-prominent. (**H**) Longitudinal section of the anther. Note the absence of a secretory cavity between the two thecae. Scale bars: (**A**), 200 μm; (**B**–**H**), 100 μm.

The Angylocalyceae subclade species (Figure 4 and Figure 5) have flowers with a papilionaceous corolla and basally united stamens (*Angylocalyx)*, a non-papilionaceous corolla that consists of a large vexillum and reduced abaxial petals (*Alexa* and *Castanospermum*), a non-papilionaceous corolla that consists of a slightly wider vexillum and four equal abaxial petals and united stamens (*Xanthocercis*). A prominent anther apex occurs in the two species of *Alexa* (*Alexa grandiflora* and *A. superba*) and *Castanospermum australe* (Figure 4E,I and Figure 5C,E); a non-prominent anther apex occurs in species of *Angylocalyx*, in *Xanthocercis zambesiaca* and six species of *Alexa* (Figure 4A–C,G,H,L and Figure 5A,G).

**Figure 6:**
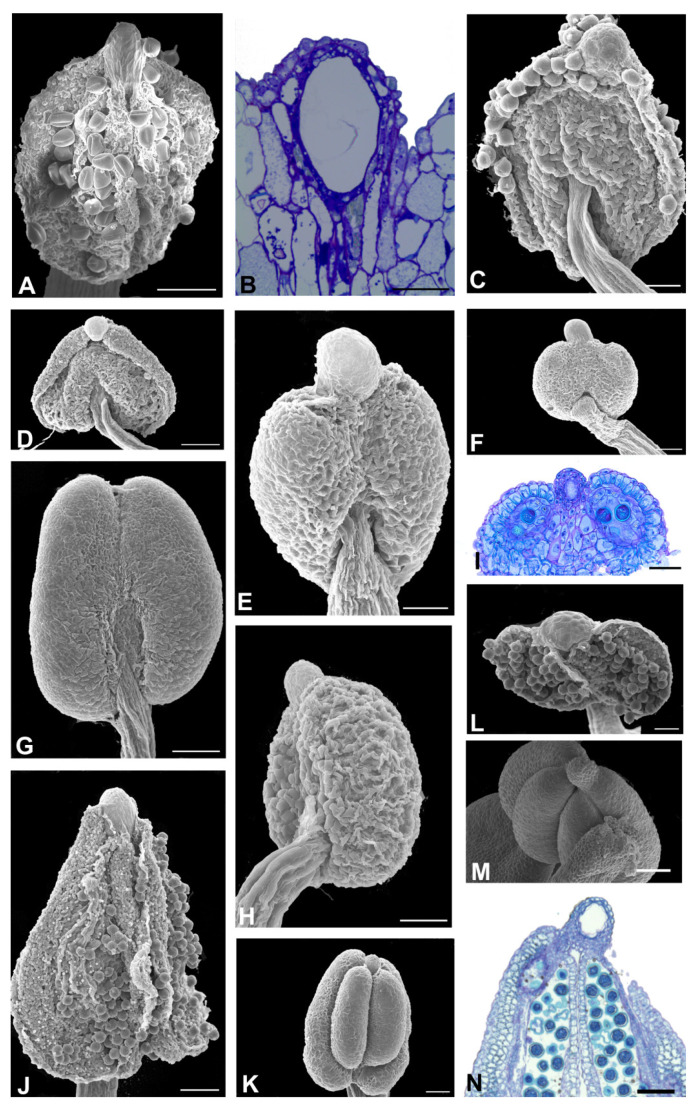
Aspects of the anther apex morphology in the species of the Dipterygeae subclade. (**A**,**C**–**H**,**J**–**M**) = SEM, (**B**,**I**,**N**) = light microscopy, LM. (**A**,**B**) *Dipteryx alata*. (**A**) Adaxial view of the anther with longitudinal dehiscence and pollen grains adhering to the anther apical region. (**B**) Longitudinal section showing the anther apex with a secretory cavity. (**C**) *D. lacunifera.* Abaxial view of the anther. Note that the anther apex is prominent. (**D**) *D. magnifica.* Abaxial view. Note that the anther apex is prominent. (**E**) *D. micrantha.* Abaxial view. Note that the anther apex is prominent. (**F**) *D. odorata.* Abaxial view of the anther. Note that the anther apex is prominent. (**G**) *D. polyphylla.* Abaxial view of the anther. Note that the anther apex is non-prominent. (**H**,**I**) *D. punctata.* H. Lateral view of the anther. Note that the anther apex is prominent. (**I**) Longitudinal section showing the anther apex with a secretory cavity with a spherical lumen. (**J**) *D. rosea.* Lateral view of the anther. Note that the anther apex is prominent. (**K**) *Pterodon abruptus*. Adaxial view of the anther. Note that the anther apex is prominent. (**L**) *P. emarginatus*. Frontal view of the anther. Note that the anther apex is prominent. (**M**,**N**) *P. pubescens*. (**M**) Lateral view of the anther. Note that the anther apex is prominent. (**N**) Longitudinal section showing the anther apex with a secretory cavity. Scale bars (**A**,**D**,**F**,**G**,**J**,**K**,**M**), 100 μm; (**B**), 20 μm; (**C**,**E**,**I**,**L**,**H**), 50 μm; (**N**), 200 μm.

**Figure 7:**
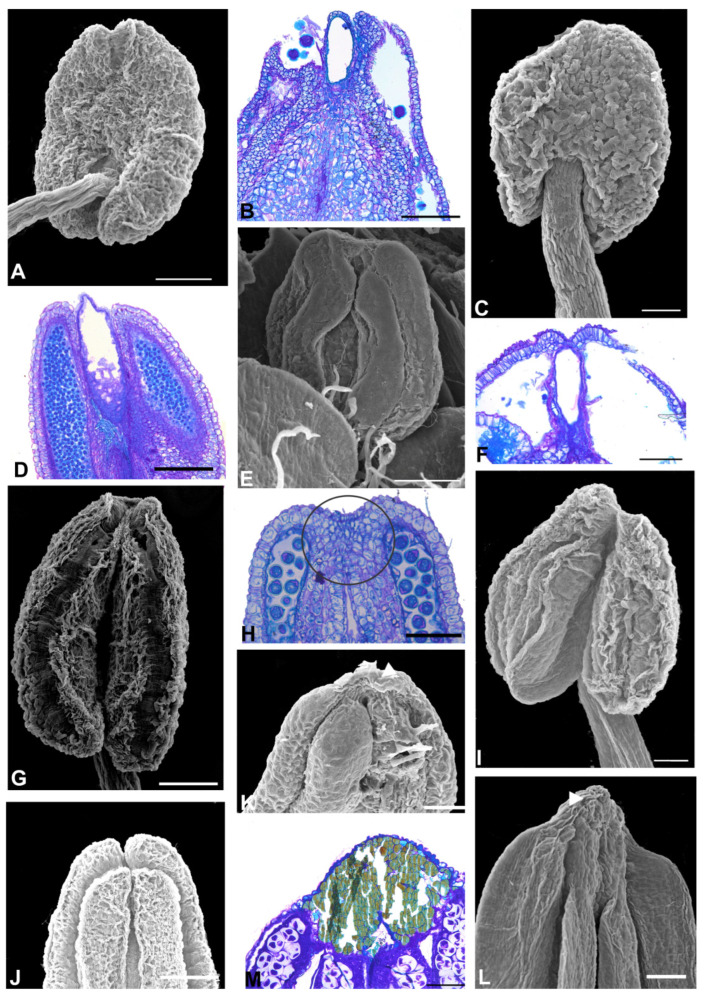
Aspects of the anther apex morphology in the species of the Dipterygeae subclade. (**A**,**C**,**E**,**G**,**I**–**L**) = SEM, (**B**,**D**,**F**,**H**,**M**) = light microscopy, LM. (**A**,**B**) *Taralea. cordata.* (**A**) Abaxial view of the anther. Note that the anther apex is non-prominent. (**B**) Longitudinal section showing the anther apex with a secretory cavity with an oval lumen. (**C**,**D**) *T. crassifolia*. (**C**) Abaxial view of the anther. Note that the anther apex is non-prominent. (**D**) Longitudinal section showing the anther apex with a secretory cavity with an oval lumen. (**E**,**F**) *T. nudipes*. (**E**) Adaxial view of the anther. Note that the anther apex is non-prominent. (**F**) Longitudinal section showing the anther apex with a secretory cavity with an oval lumen. (**G**,**H**) *T. oppositifolia.* (**G**) Adaxial view of the anther. Note that the anther apex is non-prominent. (**H**) Longitudinal section showing parenchymal cells (circle) of the anther. (**I**) *T. reticulata*. Adaxial view of the anther. Note that the anther apex is non-prominent. (**J**) *T. rigida.* Adaxial view of the anther. Note that the anther apex is non-prominent. (**K**) *Monopteryx inpae.* Lateral view of the anther. Note that the anther apex is non-prominent (arrowhead). (**L**,**M**) *M. uaucu*. (**L**) Lateral view of the anther. Note that the anther apex is prominent (arrowhead). (**M**) Longitudinal section showing the anther apex with phenolic cells. Scale bars: (**A**,**E**–**I**,**K**–**M**), 100 μm; (**B**,**D**,**J**), 200 μm; (**C**). 50 μm.

The Dipterygeae subclade species (Figure 6 and Figure 7) have flowers with a papilionaceous corolla formed by a vexillum, two wings, and two keels, and a monadelphous androecium (*Dipteryx*, *Pterodon,* and *Taralea*); and a non-papilionaceous corolla that consists of a vexillum, two reduced wings, connate and open keels exposing the free stamens (*Monopteryx*). A prominent anther apex occurs in seven species of *Dipteryx*, all species of *Pterodon* and *Monopteryx uaucu* (Figure 6A,C,D–F,H,J–M and Figure 7L); a non-prominent anther apex occurs in *Dipteryx polyphylla*, in all species of *Taralea* and *Monopteryx inpae* (Figure 6G and Figure 7A,C,E,G,I–K).

**Figure 8:**
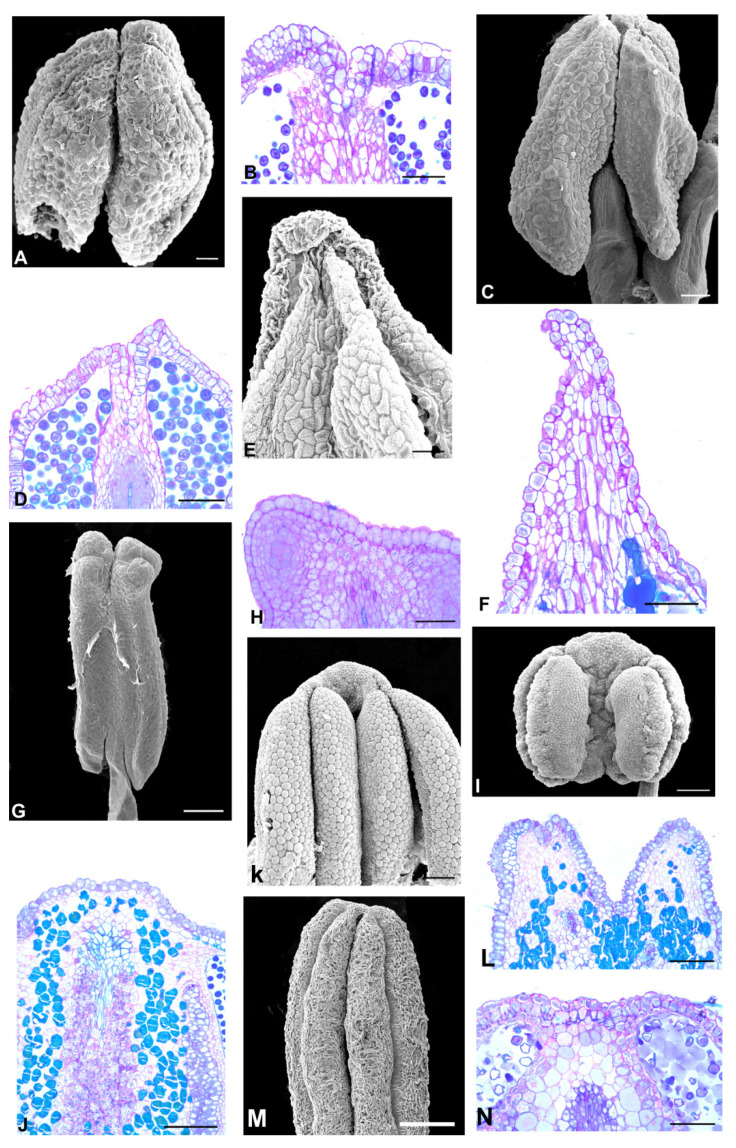
Aspects of the anther apex morphology in the species of the *Ateleia, Candolleodendron, Cyasthostegia, Swartzia, Uleanthus.* (**A**,**C**,**E**,**G**,**K**,**I**,**M**) = SEM, (**B**,**D**,**H**,**F**,**J**,**L**,**N**) = light microscopy, LM. (**A**,**B**) *Ateleia glazioveana.* (**A**) Adaxial view of the anther. Note that the anther apex is non-prominent. (**B**) Longitudinal section showing parenchymal cells at the anther apex. Note that there is no secretory cavity between the anther’s thecae. (**C**,**D**) *Ateleia guaraya.* (**C**) Adaxial view of the anther. Note that the anther apex is non-prominent. (**D**) Longitudinal section showing parenchymal cells at the anther apex. (**E**,**F**) *Candolleodendron brachystachyum.* (**E**) Adaxial view of the anther. Note that the anther apex is prominent. (**F**) Longitudinal section showing parenchymal cells at the anther apex. (**G**,**H**) *Cyathostegia mathewsii.* (**G**) Abaxial view of the anther. Note that the anther apex is non-prominent. (**H**) Longitudinal section showing parenchymal cells of the anther. (**I**,**J**) *Swartzia langsdorffii* (**I**) Adaxial view of the anther of the small stamens with a small prominence apex. (**J**) Longitudinal section showing the anther apex with phenolic cells. (**K**) Adaxial view of the anther of the large stamens with a small prominent apex. (**L**) Longitudinal section showing the anther apex with phenolic cells. (**M**,**N**) *Uleanthus erythrinoides*. (**M**) Adaxial view of the anther. Note that the anther apex is non-prominent. (**N**) Longitudinal section showing parenchymal cells of the anther. Scale bars: (**A**–**G**,**I**,**J**,**N**), 100 μm; (**H**), 50 μm; (**L**,**M**), 200 μm.

The additional species (Figure 8), *Ateleia glazioveana*, *A. guaraya*, *Cyathostegia mathewsii*, and *Uleanthus erythrinoides*, exhibit non-prominent anther apices (Figure 8A,C,G,M). In contrast, *Candolleodendron brachystachyum* and *Swartzia langsdorffii* have prominent anther apices (Figure 8E,K,I).

### 2.2. Anther Glands

The anthers of the ADA clade species have three types of glands: secretory cavity, secretory duct, and phenolic idioblast (Table 1).

A secretory cavity occurs embedded in the anther apex of *Cordyla*, *Myrocarpus*, *Myrospermum* species (Amburaneae subclade), of all species of *Dipteryx*, *Pterodon,* and of three species of *Taralea* (*T. cordata*, *T. crassifolia* and *T. nudipes*) (Dipterygeae subclade) (Figure 2H, Figure 3C,F,H, Figure 6B,I,N and Figure 7B,D,F). The lumen shape varies from spherical to oval between *Dipteryx* and *Taralea* (compare Figure 6B and Figure 7B).

A secretory duct occurs embedded in the anther apex and extends distally between thecae of the *Myroxylon* species (Amburaneae subclade) (Figure 3K). The lumen shape is elongated (Figure 3K).

In the remaining species, the anther apex does not show a secretory cavity or a secretory duct (Figure 2B,E,O, Figure 3B,M, Figure 4D,F,J,L, Figure 5B,D,F,H, Figure 7H,M and Figure 8B,D,H,F,J,L,N).

Phenolic idioblasts are found in the anther apex of *Petaladenium*, six *Alexa* species, two *Monopteryx* species, and in *Swartzia langsdorffii* (Figure 3M, Figure 4D,F,L, Figure 7M and Figure 8J,L). These species do not have cavities or ducts.

### 2.3. Distribution of Secretory Cavities in the Anther of the Amburaneae and Dipterygeae Subclades

**Figure 9:**
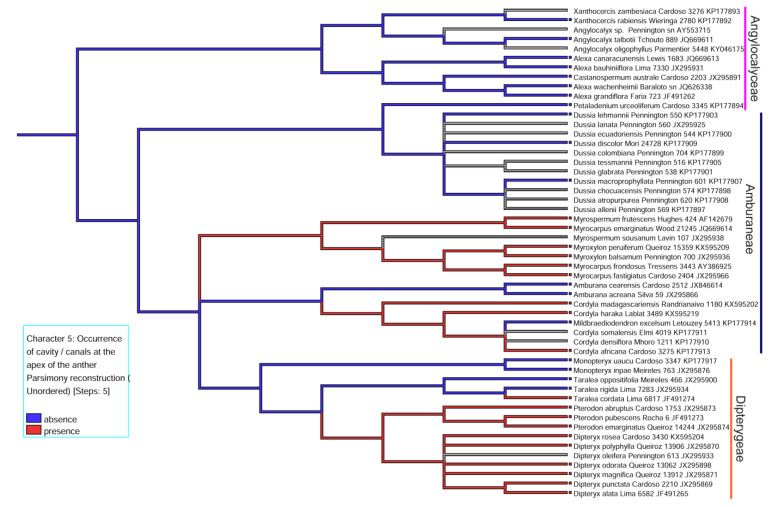
Representation of the modified LPGW cladogram (2017) for the ADA clade, showing the reconstruction of the character history in the occurrence of glands in anthers of the group. The red color represents the presence of glands in the lineage, the blue color represents the absence of glands, and the gray color represents doubt about the occurrence of the glands. The reconstruction indicates that the presence of glands has two plausible hypotheses: the appearance of the glands in the subclades (Amburaneae and Dipterygeae) or their loss in some representatives of Amburaneae and Dipterygeae and in all representatives of Angylocalyceae.

By tracing the evolutionary history of the character “occurrence of secretory cavity/duct in the anther”, it was inferred that the presence of cavity/duct in the anther was acquired in most representatives of the Dipterygeae subclade and some of the Amburaneae subclade (Figure 9).

### 2.4. Correlations between Character -States

**Figure 10:**
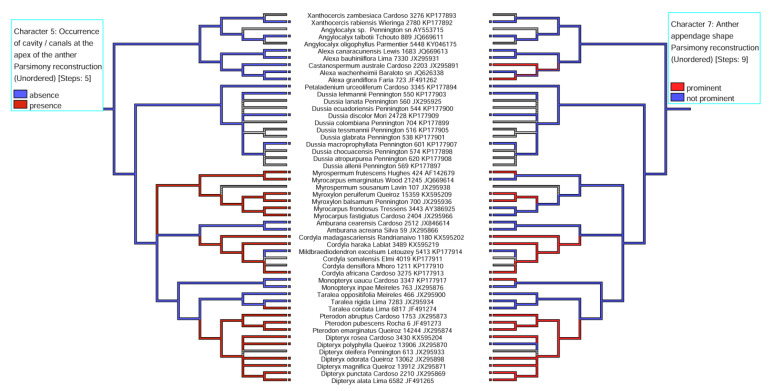
Modified LPGW cladogram (2017) for the ADA clade showing the mirroring of the character state reconstruction for “secretory cavity/duct occurrence” vs. “shape of the anther apex”.

The pairings of the character -state reconstructions related to the occurrence of cavity/duct at the anther apex vs. shape of the anther apex indicate a positive correlation between these two characters (Figure 10). However, it is noteworthy that in 5 of the 20 species (*Myrocarpus emarginatus*, *M. fastigiatus*, *M. frondosus*, *Dipteryx polyphylla*, and *Taralea cordata*), this correlation was negative that is, even without a prominent anther apex, there was a secretory cavity in the anther.

**Figure 11:**
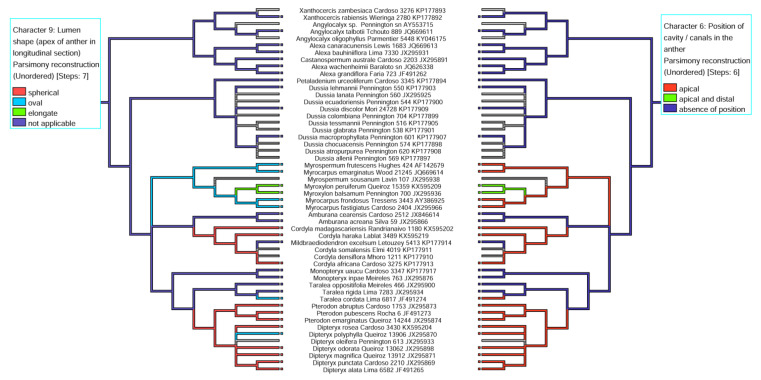
Modified LPGW cladogram (2017) for the ADA clade showing the mirroring of the character state reconstruction for “lumen shape” vs. “position of the secretory cavity/duct in the anther”.

The pairings of the character-state reconstructions related to the shape of the lumen verified at the anther apex in longitudinal section vs. position of the cavity/duct in the anther indicate a positive correlation between elongated shape and apical and distal position (*Myroxylon balsamum* and *M. peruiferum*), spherical shape and apical position (*Cordyla africana*, *C. madasgascariensis*, *C. haraka*, *Dipteryx alata*, *D. magnifica*, *D. rosea*, *D. odorata*, *D. punctata*, *Pterodon abruptus*, *P. emarginatus*, *P. pubescens*), and oval shape and apical position (*D. polyphylla*, *Myrocarpus emarginatus*, *M. fastigiatus*, *M. frondosus*, *Myrospermum frutescens*, *Taralea cordata*) (Figure 11).

## 3. Discussion

Our study highlights how glandular appendages occur in the anther of the ADA clade and provides an opportunity to clarify their enigmatic evolutionary history within early-branching papilionoids. The glandular appendage in the anther has been previously reported in the *Dipteryx alata* and *Pterodon pubescens* species of the Dipterygeae subclade [7,13], and also occurring in the species of the Amburaneae subclade.

### 3.1. Distribution and Location of Secretory Cavities/Ducts

The large ADA clade comprises morphologically eclectic genera with a diverse occurrence and structure of a glandular appendage in the anther, and the shape and location of this gland in the anther. Among 50 species analyzed within the ADA clade, 21 exhibit secretory cavities, two secretory ducts, and nine phenolic idioblasts, for a total of 64% species with a secretory structure in the anther apex.

Anatomical analyses of the anther in a longitudinal section showed that in most species analyzed, the gland is a secretory cavity with lumen shapes ranging from spherical to oval (see Table 1). *Myroxylon balsamum* and *M. peruiferum*, two species of the Amburaneae subclade, are the exceptions. The gland in the anther exhibits an elongated lumen so that, the term, secretory duct, becomes more appropriate. Variations in the lumen shape and, consequently, the difficulties generated in the typification of the gland, have been extensively explored in the literature, especially in studies with the leaf [15,20] and the stem [14,15,18,19].

The glandular appendage in the anther is most evident in the species of *Pterodon*, *Dipteryx* [7,13], present study, and *Cordyla*, which have secretory cavities with a spherical lumen, except for *D. polyphylla* in which the appendix is not very prominent, and the cavities have oval lumens. Similarly, *Taralea cordata*, *T. crassifolia*, and *T. nudipes* exhibit non-prominent anther appendages and contain a secretory cavity with an oval lumen, which is found in the region between the two thecae. In these species, the lumen of the cavity has the same shape as described for the vegetative organs of *Taralea oppositifolia* [15].

It is interesting to note that the secretory cavity located at the apex of the anther is subepidermal, and the epidermis cells have phenolic compounds see [13]. In *Monopteryx* and some species of *Alexa*, the cells of the anther appendix also exhibit phenolic content, although they do not have a secretory cavity. The appendix composed of phenolic cells that the anthers exhibit must be related to the floral structure, which is non-papilionaceous in the species of *Alexa* and *Monopteryx*. In these cases, the wing petals are reduced, the keel petals are united and opened, and the free stamens are exposed. The presence of phenolic compounds in the anther apex may be associated with the defense against herbivory or UV radiation since anthers are not protected by the petals as in a papilionaceous flower [34,35,36,37].

An interesting fact is the association between a secretory cavity in the anther and the leaf. Secretory cavities are present at the anther apex of closely related species such as *Cordyla africana*, *C. haraka*, *C. madagascariensis*, *Myrocarpus emarginatus*, *M. fastigiatus, M. frondosus*, and *Myrospermum frutescens.* In contrast, *Myroxylon balsamum*, and *M. peruiferum* exhibit a secretory duct at the anther apex. The secretory cavity or duct is absent in the species of *Amburana*, *Dussia*, *Mildbraediodendron*, and *Petaladenium* (Amburaneae subclade), and in the species of *Alexa*, *Angylocalyx*, *Castanospermum,* and *Xanthocercis* (Angylocalyceae subclade). The presence of a secretory cavity and ducts in the leaflet is shared by species of *Myrocarpus* (*M. emarginatus*, *M. fastigiatus*, *M. frondosus*, *M. leprosus, M. venezuelensis*), *Myrospermum* (*M. frutescens*, *M. sousanum*), *Myroxylon* (*M. peruiferum* and *M. balsamum*) and *Cordyla* (*C. haraka*, *C. africana* and *C. madagascariensis* [20,21] which demonstrates that these structures also occur in the floral organs of these species.

### 3.2. Evolutionary History of the Presence of a Secretory Cavity in the Anther of the ADA Clade

Our hypotheses were postulated to explain the occurrence of glands in anthers of the ADA clade species and have two robust explanations: (1) the appearance of the anther glands in the Amburaneae and Dipterygeae subclades or (2) their loss in some species of Amburaneae and Dipterygeae and all species of Angylocaleceae (see Figure 9).

Considering that the secretory cavities are present in other genera of the Amburaneae subclade, it is concluded that they are not a synapomorphy of the Dipterygeae subclade, as suggested by Leite et al. [7].

In the Dipterygeae subclade, our data suggest that secretory cavities may have been acquired in *Dipteryx* + *Pterodon* and some species of *Taralea*. A phenolic glandular appendix may have been acquired in Monopteryx, a sister group of *Dipteryx*, *Pterodon*, and *Taralea* (see distribution in Figure 9). The glandular appendix with a phenolic epidermis in *Dipteryx*, *Pterodon*, and *Taralea* could be a remnant of the *Monopteryx* phenolic appendage.

The Amburaneae subclade is remarkable because of its high level of floral diversity, production of coumarins (*Amburana*), red resin from bark and twigs (*Dussia*), balsams (*Myrocarpus*, *Myrospermum*, *Myroxylon*), and punctate glandular leaves of several genera (*Cordyla*, *Mildbraediodendron*, *Myrocarpus*, *Myrospermum*, and *Myroxylon)* [27,38,39]. The presence of glands at the anther apex is also noteworthy. Our data suggest that secretory cavities/ducts may have been acquired in the well-supported clades *Myroxylon* + *Myrocarpus* (non-papilionaceous flowers) and *Myrospermum* (papilionaceous flower). Another interesting fact is the presence of a secretory duct only in *Myroxylon*, and therefore, an autapomorphy of *Myroxylon*. Although *Cordyla* and *Mildbraediodendron* have a swartzioid-like floral morphology [12,38], the flowers have an entire calyx, no petals, and numerous free stamens, only *Cordyla* exhibits a secretory cavity in the anther apex. In contrast, the genus *Amburana*, sister to *Cordyla* + *Mildbraediodendron*, does not have a secretory cavity in the anther and has a one-petalled corolla and 10 free stamens. The absence of a secretory cavity in the anther apex of the genera *Petaladenium* and *Dussia* reflects their positioning in the phylogenetic tree as sister genera. They also present a papilionaceous corolla and basally united stamens [10]. A peculiar characteristic of *Petaladeninum urceoliferum* is its wing petals with glands, while in *Dussia* (its sister genus), the glands are found on the bract and bracteoles [9], although there are no anatomical studies on the composition of these structures.

In the Angylocalyceae subclade, our data suggest that secretory cavities were not acquired in the genera *Alexa*, *Angylocalyx*, *Castanospermum*, and *Xanthocercis*, defining them as a sister group of the Amburaneae and Dipterygeae subclades. An appendix producing phenolic compounds may have been acquired in some *Alexa* species, a sister group of *Castanospermum*, both with similar floral morphology, non-papilionaceous corolla, and 10 free stamens [12]. *Angylocalyx* and *Xanthocercis*, sister genera, exhibit a distinct floral morphology [12].

Other relevant data are the occurrence of secretory cavity/duct vs. anther appendage shape (see Figure 10) and lumen shape vs. position of cavity/duct in the anther (see Figure 11). It is likely that the anthers with a prominent apex also exhibit secretory cavities with a spherical lumen, different from those with a non-prominent apex, which exhibit secretory cavities more internalized in the anther, between the thecae, with the lumen being oval. Thus, we suppose that the shape of the anther is related to the lumen shape in species with a secretory cavity in the anther (see Figure 10 and Figure 11). Another interesting result is the lumen shape in the clades *Myrocarpus* + *Myrospermum*, and *Myroxylon*. *Myrocarpus*+ *Myrospermum* exhibit secretory cavities more internalized in the anther between the thecae, with an oval lumen and a prominent/non- prominent apex, respectively, different from *Myroxylon,* which exhibits a secretory duct with a rounded lumen in its apical portion and is elongated in the lower portion between the thecae following the shape of the anther apex.

The secretory cavities of *Dipteryx* (except *D. polyphylla*) and *Pterodon* are anatomically more similar to each other than to those of *Taralea cordata*, *T. crassifolia* and *T. nudipes*, confirming previous data obtained about the flower [7], leaf, stem [15] and leaflets [16] and corroborating the phylogeny data (see LPGW [1]). This fact reflects the topology of a phylogenetic tree in the subclades, which places them as sister groups see [1,12,22].

The inclusion of other species with a non-papilionaceous corolla from the Swartzieae clade (*Ateleia glazioveana*, *A. guaraya*, *Candolleodendron brachystachyum*, *Cyathostegia mathewsii*, *Swartzia langsdorffii*) and genistoid clade (*Uleanthus erythrinoides*) [1] suggests that the presence of secretory cavities/ducts in the anther apex may be restricted to the members of the subclades Amburaneae and Dipterygeae. There are no reports on cavities secretory/duct in the anthers apex of other papilionoid legumes. *Hymenaea verrucosa* Gaertn. (subfamily Detarioideae) exhibits a secretory cavity in the connective region (ventral region) of the anthers [40]; and in *Stryphnodendron adstringens, Tetrapleura tetraptera*, *Adenanthera pavonine,* and *Pentaclethra macroloba* (Mimosoid clade, subfamily Caesalpinioideae) such anther glandular appendages can comprise secretory emergences [41].

### 3.3. Evolutionary Significance of the Corolla, Type of Androecium vs. Presence of a Secretory Cavity/Duct in the Anther

Our data plotted in phylogeny have not yet made it possible to correlate the type of corolla and androecium with a secretory cavity/duct in the anther. The presence of a secretory cavity/duct has been reported in species with apetalous, non-papilionaceous flowers with numerous free stamens, including *Cordyla africana*, *C. haraka* and *C. madagascariensis*. *Myrocarpus emarginatus*, *M. fastigiatus,* and *M. frondosus* exhibit five undifferentiated petals with 10 basally united stamens, *Myrospermum frutescens* exhibits a papilionaceous corolla with 10 free stamens; *Myroxylon balsamum* and *M. peruiferum* exhibit a non-papilionaceous corolla with the widely oval banner and 10 free stamens. Therefore, a correlation with pollination seems plausible.

In *Dipteryx alata* and *Pterodon pubescens*, species of the Dipterygeae subclade, the anther glands consist of a cavity secreting sticky substances (oleoresins and polysaccharides) that play a key role during the flower’s lifespan by aggregating pollen grains and attaching them to the floral visitor’s body, besides maximizing the pollen release mechanism that is intermediate between the valvular and the explosive [13]. The same mechanism probably occurs in *Myrospermum frutescens* because these species are pollinated by insects [42]; *Myrocarpus frondosus* has a non-papilionaceous corolla but is pollinated by insects [43], probably aggregating pollen grains.

In *Myroxylon peruiferum*, the ducts secrete a substance that aggregates pollen grains; however, when in contact with air, the resinous content secreted together with the pollen grains hardens, probably acting on pollination by sunbirds (personal observation). The same must occur in *Cordyla africana*, which has petaliferous flowers rich in nectar that are pollinated by sunbirds [44].

Species with phenolic idioblasts at the anther apex, such as those of *Alexa* and *Monopterx*, are ornithophilous [27] and entomophilous [45], respectively. There are also reports of sphingophily [46,47] and chiropterophily [48] for *Alexa*. The lack of information on the pollination biology for these species makes it difficult to understand the function of these glands in early-diverging papilionoids.

However, analyzing the corolla shape and the presence of a secretory structure in the anther concerning the pollinator (insect or bird), probably the corolla type associated with the material exuded by the secretory structure at the anther apex at different proportions found in each species acts by favoring the different pollinators.

### 3.4. Outlook

We studied the evolution of the anther’s glandular appendage in early-diverging papilionoid genera, reporting this condition in a large number of species.

The diversity of the subfamily Papilionoideae expressed in terms of floral morphology, such as loss of petals, undifferentiated petals, numerous free stamens, entire calyx in bud, and radially symmetric flowers [5,8,49,50,51,52,53,54,55] is particularly common among the early-diverging papilionoid genera [5,6,7,8,9,10]. Additionally, secretory cavities/ducts in the anther in some species of the Dipterygeae and Amburaneae subclades [7,13], present study, is a unifying character-state for these groups. This condition has not been previously reported for representatives of the most recent divergent papilionoids.

## 4. Materials and Methods

### 4.1. Sampling Plant Material

We examined at least one species in each genus of the Amburaneae (*Amburana*, *Cordyla*, *Dussia*, *Mildbraediodendron*, *Myrocarpus*, *Myrospermum*, *Myroxylon,* and *Petaladenium*), Angylocalyceae (*Alexa*, *Angylocalyx*, *Castanospermum*, and *Xanthocercis*), and Dipterygeae subclades (*Dipteryx*, *Pterodon,* and *Monopteryx*). In addition, we included six species with non-papilionoid flowers outside the ADA clade to map the character of other distant taxa (Table S1).

Samples of flowers and floral buds (1 or/and 2) were obtained from herbarium specimens (Table S1) and treated with 2% KOH solution for 2 h, washed several times in distilled water [56], and stored in 70% ethanol. The anthers were removed and prepared for observations by scanning electron microscopy (SEM) and light microscopy (LM).

### 4.2. Scanning Electron Microscopy

For SEM analysis, anthers were critical point dried in a Balzers CPD 030 dryer (Balzers, Liechtenstein), mounted on aluminum stubs with colloidal carbon, coated with gold in a Bal Tec SCD 050 sputter coater, and observed with a Jeol JSM 6610LV scanning electron microscope (Tokyo, Japan).

### 4.3. Light Microscopy

For LM analysis, anthers were embedded in historesin [57] and longitudinally sectioned (2–3 μm thick) with a rotary microtome (Leica RM 2245, Wetzlar, Germany). Sections were stained with 0.05% toluidine blue in phosphate buffer, pH = 6.8 [58] and photographed with a light microscope (Leica DM5000 B) coupled to a digital camera (Leica DFC295).

### 4.4. Phylogenetic Analysis

The evolution of the glands in anthers from the ADA clade was investigated based on a recent phylogenetic hypothesis [1]. The characters selected to compose the data matrix were the absence or presence of secretory cavity/ducts, anther appendage shape (prominent, not prominent), anther appendix position (apical, distal, apical/distal), lumen shape (spherical, oval, elongated), and absence or presence of phenolic compound at the apex of the anther (Table 2). We used the Mesquite program [59] to map the selected characters (Table 2) on the RAXML tree obtained by the Legume Phylogeny Working Group (LPWG) [1]. For this purpose, we chose “trace character history”, selecting the option “parsimony ancestral states”.

## Figures and Tables

**Table 1:** Matrix of morphological data obtained from information in the literature and analysis of herbarium material for the characters selected in the present study. Empty cells mean missing information.

Subclade	Species	Number of Sepals	Number of Petals	Number of Stamens	Stamen Connation	Occurrence of Cavity/Canal at the Anther	Position of Cavity/Canal at the Anther	Shape of Lumen of Cavity/Canal	Shape of the Anther Apex	Occurrence of Phenolic Cells at the Anther Apex	References
Amburaneae	*Amburana acreana* (Ducke) A.C. Sm.	5	1	10	free	absent				absence	[23], Present study
	*Amburana cearensis* (Allemão) A.C. Sm.	5	1	10	free	absent				absence	[7]
	*Amburana erythrosperma* E. P. Seleme, C. H. Stirt. & V. F. Mansano	5	1	10	free	absent				absence	Present study
	*Cordyla africana* Lour	3	0	numerous		cavity	apical	spherical	prominent		Present study
	*Cordyla haraka* Capuron	3	0	numerous	free	cavity	apical	spherical	prominent		Present study
	*Cordyla madagascariensis* R. Vig.	3	0	numerous	free	cavity	apical	spherical	prominent		Present study
	*Dussia discolor* (Benth.) Amshoff	5	5	10	fused at the base	absent			not prominent	absence	[6], Present study
	*Dussia lehmannii* Harms	5	5	10	fused at the base	absent			not prominent	absence	[24], Present study
	*Dussia macroprophyllata* (Donn. Sm.) Harms	5	5	10	fused at the base	absent			not prominent	absence	[24], Present study
	*Dussia martinicensis* Krug & Urb. ex Taub	5	5	10	fused at the base	absent			not prominent	absence	[24], Present study
	*Dussia tessmannii* Harms	5	5	10		absent			not prominent	absence	Present study
	*Mildbraediodendron excelsum* Harms	5	0	numerous	free	absent			not prominent	absence	[25,26], Present study
	*Myrocarpus emarginatus* A.L.B. Sartori & A.M.G. Azevedo	5	5	10	fused at the base	cavity	apical	oval	not prominent		[27,28], Present study
	*Myrocarpus fastigiatus* Allemão	5	5	10	fused at the base	cavity	apical	oval	not prominent		[27,28], Present study
	*Myrocarpus frondosus* Allemão	5	5	10	fused at the base	cavity	apical	oval	not prominent		[20,27], Present study
	*Myrospermum frutescens* Jacq	5	5	10	free	cavity	apical	oval	prominent		[27,29], Present study
	*Myroxylon balsamum* (L.) Harms	5	5	10	free	duct	apical, distal	elongate	prominent		[5,27], Present study
	*Myroxylon peruiferum* L. f.	5	5	10	free	duct	apical, distal	elongate	prominent		[27], Present study
	*Petaladenium urceoliferum* Ducke	5	5	10	(fused at the base = nearly free)	absent			not prominent	absence	[9], Present study
Angylocalyceaee	*Alexa bauhiniiflora* Ducke	5	5	8/10	free	absent			not prominent	presence	[30], Present study
	*Alexa canaracunensis* Pittier	5	5	10	free	absent			not prominent	presence	[30], Present study
	*Alexa cowanii* Yakovlev	5	5	10	free	absent			not prominent	presence	[30], Present study
	*Alexa grandiflora* Ducke	5	5	10	free	absent			prominent	presence	[30], Present study
	*Alexa imperatricis* (R.H. Schomb.) Baill.	5	5	8/10	free	absent			not prominent	absence	[30], Present study
	*Alexa leiopetala* Sandwith	5	5			absent			not prominent	presence	
	*Alexa superba* R.S. Cowan	5	5	10/15	free	absent			prominent	absence	[30], Present study
	*Alexa wachenheimii* Benoist	5	5	10	free	absent			not prominent	presence	
	*Angylocalyx pynaertii* De Wild.	5	5	10	monadelphous	absent			not prominent	absence	[31], Present study
	*Angylocalyx talbotii* Hutch. & Dalziel	5	5	10	monadelphous	absent			not prominent	absence	Present study
	*Castanospermum australe* A. Cunn. ex Mudie	5	5	10	free	absent			prominent	absence	[5], Present study
	*Xanthocercis zambesiaca* (Baker) Dumaz-le-Grand	5	5	10	fused at the base	absent			not prominent	absence	[32], Present study
Dipterygeae	*Dipteryx alata* Vogel	5	5	10	monadelphous	cavity	apical	spherical	prominent		Present study
	*Dipteryx lacunifera* Ducke	5	5	10	monadelphous	cavity	apical	spherical	prominent		Present study
	*Dipteryx magnifica* (Ducke) Ducke	5	5	10	monadelphous	cavity	apical	spherical	prominent		Present study
	*Dipteryx micrantha* Harms	5	5	10	monadelphous	cavity	apical	spherical	prominent		Present study
	*Dipteryx odorata* (Aubl.) Willd.	5	5	10	monadelphous	cavity	apical	spherical	prominent		Present study
	*Dipteryx polyphylla* (Huber) Ducke	5	5	10	monadelphous	cavity	apical	oval	not prominent		Present study
	*Dipteryx punctata* (Blake) Amshoff	5	5	10	monadelphous	cavity	apical	spherical	prominent		Present study
	*Dipteryx rosea* Spruce ex Benth	5	5	10	monadelphous	cavity	apical	spherical	prominent		Present study
	*Pterodon abruptus* (Moric.) Benth.	5	5	10	monadelphous	cavity	apical	spherical	prominent		Present study
	*Pterodon emarginatus* Vogel	5	5	10	monadelphous	cavity	apical	spherical	prominent		Present study
	*Pterodon pubescens* (Benth.) Benth.	5	5	10	monadelphous	cavity	apical	spherical	prominent		Present study
	*Taralea cordata* Ducke	5	5	10	monadelphous	cavity	apical	oval	not prominent		Present study
	*Taralea crassifolia* (Benth.) Ducke	5	5	10	monadelphous	cavity	apical	oval	not prominent		Present study
	*Taralea nudipes* (Tul.) Ducke	5	5	10	monadelphous	cavity	apical	oval	not prominent		Present study
	*Taralea oppositifolia* Aubl.	5	5	10	monadelphous	absent			not prominent		Present study
	*Taralea reticulata* (Benth.) Ducke.	5	5	10	monadelphous	absent			not prominent		Present study
	*Taralea rigida* Schery	5	5	10	monadelphous	absent			not prominent		Present study
	*Monopteryx inpae* W.A.Rodrigues	5	5	10	free	absent	apical		not prominent	presence	[10,33], Present study
	*Monopteryx uaucu* Spruce ex Benth.	5	5	10	free	absent	apical		prominent	presence	[10,33], Present study
Outgroup	*Ateleia glazioveana* Baill.	5	1	10	free	absent			not prominent	absence	Present study
	*Ateleia guaraya* Herzog	5	1	10	free	absent			not prominent	absence	Present study
	*Candolleodendron brachystachyum* (DC.) R.S. Cowan	5	1	numerous	free	absent			prominent	absence	Present study
	*Cyathostegia mathewsii* (Benth.) Schery	5	1	numerous	free	absent			not prominent	absence	Present study
	*Swartzia langsdorffii Raddi*	4	0	2–3 (smaller stamens), numerous (larger stamens)	free	absent			prominent	presence	Present study
	*Uleanthus erythrinoides* Harms	5	4	10	free	absent			not prominent	absence	Present study

**Table 2:** Morphological characteristics evaluated in taxa of the Amburaneae, Angylocaleceae, Dipterygeae subclades, *Ateleia*, *Candolleodendron*, *Cyathostegia*, *Swartizia* and *Uleanthus*.

Variables	
1. Number of sepals on the mature flower	(0) five, (1) four, (2) three
2. Number of petals on the mature flower	(0) zero, (1) one, (2) four, -(3) five
3. Number of stamens	(0) up to 10, (1) 10, -(3) numerous (= over 10)
4. Type of stamen connation	(0) free, (1) fused at the base, (2) monadelphous
5. Occurrence of cavity /canals at the apex of the anther	(0) absence, (1) presence
6. Position of cavity /canals in the anther	(0) apical, (1) distal, (2) apical and distal, (3) absence of position
7. Anther appendage shape	(0) prominent, (1) not prominent
8. Phenolic compound tissue at the apex of the anther	(0) absence, (1) presence
9. Lumen shape (apex of anther in longitudinal section)	(0) spherical, (1) oval, (2) elongate, (3) not applicable

## Data Availability

Data sharing is not applicable to this article.

## Supplementary Materials

The following supporting information can be downloaded at: https://www.mdpi.com/article/10.3390/plants11070835/s1, Table S1: Species of Amburaneae, Angylocalyceae, Dipterygeae subclades and outgroup. IAN = Herbário da Embrapa Amazônia Oriental; INPA = Herbário do Instituto Nacional de Pesquisas da Amazônia; KEW = Herbarium Royal Botanic Gardens; MNHN = Muséum national d’Histoire naturelle; MG = Herbário do Museu Paraense Emílio Goeldi; NYBG = The New York Botanical Garden; RB = Herbário do Jardim Botânico do Rio de Janeiro; RBSpirit = Herbário do Jardim Botânico do Rio de Janeiro; SPFR = Herbário do Departamento de Biologia da Faculdade de Filosofia Ciências e Letras de Ribeirão Preto da Universidade de São Paulo; UEC = Herbário da Universidade Estadual de Campinas; US (Smithsonian Institution).
