# Supplementary material for: Evolution of the Anther Gland in Early-Branching Papilionoids (ADA Clade, Papilionoideae, Leguminosae)
Source: Plants (Basel). 2022 Mar 22;11(7):835. doi: 10.3390/plants11070835 (PMC9002870; doi:10.3390/plants11070835)
Supplement: Supplementary file 1 [file plants-11-00835-s001.zip › plants-1617978-supplementary.pdf]

Table S1. Species of Amburaneae, Angylocalyceae, Dipterygeae subclades and outgroup.

Clade	Genera	Species	Voucher	Spirit Collection
Amburaneae	*Amburana* Schwacke & Taub.	*Amburana acreana* (Ducke) A.C. Sm.	LFG da Silva et al. 59 (RB)	
		Amburana cearensis (Allemão) A.C. Sm.	EP Seleme nº 460 (RB); NL Nunes nº 19 (RB)	
		Amburana erythrosperma E. P. Seleme, C. H. Stirt. & V. F. Mansano	GS Silva, VF Mansano 410 (UEC)	
	Cordyla Lour.	*Cordyla africana* Lour	J M Dalziel 8029 (US)	
		Cordyla haraka Capuron	DJ& BP Du Puy, G Rafamantanantsoa &G Schatz M375 (NYBG)	
		Cordyla madagascariensis R. Vig.	D Du Puy, G Lewis & B Schrire M570 (NYBG); S Malcomber et al. 1897 (NYBG)	
	Dussia Krug & Urb. ex Taub.	*Dussia discolor* (Benth.) Amshoff	J M Pires 51302 (US)	
		Dussia lehmannii Harms	J Cuatrecasas, L Willard 26094(US)	
		Dussia macroprophyllata (Donn. Sm.) Harms	D Sanchez. C Órrego, S Sylya, G Martínez, D Restrepo, J Betancur, F Roldan 1130 (NYBG)	
		Dussia martinicensis Krug & Urb. ex Taub	S R Hill 25854 (NYBG)	
		Dussia tessmannii Harms		HC Lima 5464 (RBspirit 1366)

	Mildbraediodendron Harms	*Mildbraediodendron excelsum* Harms	J Louis 1465 (MNHN)	
	Myrocarpus Allemão	*Myrocarpus emarginatus* A.L.B. Sartori & A.M.G. Azevedo	Wood et al 21248 (KEW); Wood et al 21245 (KEW)	
		*Myrocarpus fastigiatu***s** Allemão	J R Mattos, 425; M Nadruz, J Caruso (RB)	
		Myrocarpus frondosus Allemão	A M Lino 80 (RB); J Spada 181(RB)	HC LIMA 3054 (RBspirit 1410);<br>HC Lima 6905 (RBspirit1411)
	Myrospermum Jacq.	*Myrospermum frutescens* Jacq.	T. MacDougall 37 (US); Ronald Liesner 12552 & Angel González s/nº(RB)	
	Myroxylon L. f	*Myroxylon balsamum* (L.) Harms	DC Daly 8099 (INPA); DC Daly 7715 (INPA); LA de Lima 535 (INPA); LA de Lima 620 (INPA); M B Kaloo B.1065 (NYBG); W A Archer 7714 (NYBG)	
		Myroxylon peruiferum L. f.	R D Ribeiro 661, V Malioli, R Mendonça (RB)	RD Ribeiro 661 (RBspirit 1413)<br>RD Ribeiro 684 (RBspirit 1414)
	Petaladenium Ducke	*Petaladenium urceoliferum* Ducke	B G S Ribeiro 838 (RB)	
Angylocalyceae	*Alexa* Moq.	*Alexa bauhiniiflora* Ducke	GT Prance 15600 (INPA); D de AL Lima 119(INPA);	
		Alexa canaracunensis Pittier	WC Steward 235 (INPA)	
		Alexa cowanii Yakovlev	S Stephen, Tillett & L Carolyn, Tillett 45706	

			(NYBG); C Blanco 604 (NYGB);
		Alexa grandiflora Ducke	F T Kotama, PSACF-EX 0630 (RB); WA Rodrigues 7270 (INPA), WA Rodrigues 7314 (INPA), FR da Silva 159 (INPA), E de 58Lima (INPA), MFF Melo 763 (INPA), D Mitja 10158 (INPA)
		Alexa imperatricis (R.H. Schomb.) Baill.	K Kubitzki 79-199 (INPA)
		Alexa leiopetala Sandwith	M Polak & E Roberts 549 (NYBG)
		Alexa superba R.S. Cowan	GT Prance 10640 (INPA);
		Alexa wachenheimii Benoist	JM Pires 16846 (INPA)
	Angylocalyx Taub.	*Angylocalyx pynaertii* De Wild.	TB Hart 613 (NYBG)
		Angylocalyx talbotii Hutch. & Dalziel	B Satabié 313 (MNHN)
	Castanospermum A. Cunn. ex Hook.	*Castanospermum australe* A. Cunn. ex Mudie	D Cardoso 2203 (RB); R Schutz Rodrigues & JC Galvão 1262 (UEC)
	Xanthocercis Baill.	*Xanthocercis zambesiaca* (Baker) Dumaz-le-Grand	HJE Schlieben 7413 (US)
Dipyterygeae	*Dipteryx* Schreb.	*Dipteryx alata* Vogel	SPTeixeira et al. 39 (SPFR)
		Dipteryx lacunifera Ducke	JUM dos Santos Silva 628 (NYBG); R Vilhena et al 1015 (NYBG)
		Dipteryx magnifica (Ducke)	CA Cid 17, WR Buck, BW

	Ducke	Nelson, F Almeida, CDA Mota &J Lima; SS Silva s/nº (NYBG)	
	Dipteryx micrantha Harms	WA Ducke 903 (NYBG); BW Nelson 801 (NYBG)	
	Dipteryx odorata (Aubl.) Willd.	NM Lepsch da Cunha 430, E Costa Pereira, & RM Cardoso (NYBG); DN Smith & V Garcia 13864 (RB)	VFG 58 et al (RBspirit 1362)
	Dipteryx polyphylla (Huber) Ducke	Lodoño 882(NYBG); MAS Costa 749 et al. (NYBG); Dick 254 (NYBG);<br>CV Castilho 676 (INPA); CA Mackenzie 2107.701(INPA); JELS Ribeiro 1723 (INPA);	H C Lima 2787 (RBspirit 1364)
	Dipteryx punctata (Blake) Amshoff	DC Daly 5489 et al (NYBG); Wurdack & Adderloy 42844; Berti 1045 Coêlho LF s/n. (INPA);	HC Lima 7968 (RBspirit 2089)<br>HC Lima 8128 **(**RBspirit 2090)
	Dipteryx rosea Spruce ex Benth	J. M. Poole 2012 (NYBG); W.A.Ducke 902 (NYBG);<br>Silva MF da 1646 (INPA); Poole JM 2083 (INPA); Maia LA 591(INPA); Cardoso D 3430 (INPA)	HC Lima1791(RBspirit 1363)

	Pterodon Vogel	*Pterodon abruptus* (Moric.) Benth.	B. B. Klitgaard & F.C.P.Garcia 75 (NYBG); G. Martinelli 18005et al (NYBG)
		Pterodon emarginatus Vogel	K.Yamamoto 42 (SPF);F. de Barros 1173 (RB); Elder Antonio Sousa e Paiva 23 (RB); Mathes, D. D. F.; 12996 (US); J. A. Ratter 2427 (UB)
		Pterodon pubescens (Benth.) Benth.	Teixeira et al. 37 (SPFR); Teixeira et al. 38 (SPFR)
	Taralea Aubl.	*Taralea cordata* Ducke	B. Maguire, R.S. Cowan, J.J. Wurdack 29576 (NYBG); Mantovani 12710 (NYBG); R. D. Ribeiro 1280, Barbosa C.G (NYBG).
		Taralea crassifolia (Benth.) Ducke	B. Stergios 10275 (NYBG); B. Maguire s/nºC. K. Maguire (RB)
		Taralea nudipes (Tul.) Ducke	W.Rodrigues & L. Coêlho 1327 (NYBG); M. De JesusVarejão,s/n (INPA); A. P. Duarte 7255 (RB)
		Taralea oppositifolia Aubl.	JC Freitas 233 (IAN)
		Taralea reticulata (Benth.)	S.S. Tillet & C.L.Tillet

		Ducke.	45838 (NYBG)
		Taralea rigida Schery	Prance et al 28970 (NYBG); J.J.Wurdack & L.S. Adderley 43755 (NYBG)
	Monopteryx Spruce ex Benth.	*Monopteryx inpae* W.A.Rodrigues	D. Loubry 1739 (US)
		Monopteryx uaucu Spruce ex Benth.	Ducke s/nº (RB); G. Silva s/ nº (SPFR)
Outgroup		*Ateleia glazioveana* Baill.	H. C. Lima 6826 (RB)
		Ateleia guaraya Herzog	D.F. Silva 847 (RB)
		Candolleodendron brachystachyum (DC.) R.S. Cowan	R.C. Forzza 8966 (RB)
		Cyathostegia mathewsii (Benth.) Schery	B.B.Klitgaard 383 (RB)
		Swartzia langsdorffii Raddi	J. E. Meirelles s/nº (RB)
		Uleanthus erythrinoides Harms	M. Pastore, E.A.L. Afonso, A.E.S. Rocha, 999 (MG)
